# The White Clover Single-Copy Nuclear Gene *TrNAC002* Promotes Growth and Confers Drought Resistance in Plants Through Flavonoid Synthesis

**DOI:** 10.3390/plants14010031

**Published:** 2024-12-25

**Authors:** Youzhi Zhang, Wei Fu, Qi Pu, Zhirui He, Zhou Li, Lin Liu, Xiao Ma, Yan Peng

**Affiliations:** 1 College of Grassland Science and Technology, Sichuan Agricultural University, Chengdu 611130, China; b20172903@stu.sicau.edu.cn (Y.Z.); 2021302111@stu.sicau.edu.cn (W.F.); puchess@163.com (Q.P.); 2021202091@stu.sicau.edu.cn (Z.H.); zhouli2006@stu.sicau.edu.cn (Z.L.); liulinsky@126.com (L.L.); maroar@126.com (X.M.); 2 College of Life Science, Changchun Normal University, Changchun 130032, China

**Keywords:** white clover, NAC transcription factor, flavonoid, drought stress

## Abstract

White clover (*Trifolium repens*) is vulnerable to drought stress. In response to abiotic stress, plants are regulated by NAC transcription factors. The NAC in white clover has not been thoroughly documented until recently. We have identified one white clover NAC transcription factor called *TrNAC002.* TrNAC002’s coding sequence is localized to specific regions on the 3P and 5O chromosomes of white clover and is part of a single-copy nuclear gene. Subcellular localization demonstrates that TrNAC002 is located in the nucleus, while the transcriptional activity assay indicates its transcriptional activity. *Arabidopsis* plants overexpressing *TrNAC002* (OE) exhibit enlarged leaves and increased lateral root growth compared to the wild type (WT). Additionally, the expression levels of the shoot apical meristem (SAM), WUSCHEL (WUS), DNA-binding protein (DBP), and auxin-induced in root cultures3 (AIR3) genes are significantly higher in OE as compared to WT. These findings imply that TrNAC002 could promote vegetative growth by increasing the expression of these genes. Under natural drought stress, OE can survive in dry soil for a longer period of time than WT. Furthermore, OE exhibits a lower level of reactive oxygen species (ROS) level and a higher content of flavonoids than WT. This is also positively correlated with an increased flavonoid content. In white clover, the expression of *TrNAC002*, chalcone synthase (*CHS*), and chalcone isomerase (*CHI*) in leaves demonstrates significant upregulation after drought stress and ABA treatment, as does the flavonoid content. However, the pTRV-VIGS experiment suggests that pTRV2-TrNAC002 white clover shrinks compared to the Mock and Water controls. Additionally, pTRV2-TrNAC002 white clover displays a statistically higher malondialdehyde (MDA) content than the Mock and Water controls, and a significantly lower level of total antioxidant activities, flavonoid content, *CHS* and *CHI* relative expression than that of the Mock and Water controls. These findings indicate that *TrNAC002* responds to drought and modulates flavonoid biosynthesis in white clover. This study is the first to suggest that *TrNAC002* likely responds to drought via ABA and enhances plant drought resistance by synthesizing flavonoids.

## 1. Introduction

As flavonoids are specialized secondary metabolites in plants, they are present in many different types of flora. Flavonoid accumulation in a plant is often considered indicative of stress, which is why it has garnered significant interest [1]. Drought significantly raises the gene expression linked to flavonoid production [2,3,4] along with flavonoid content, demonstrating a means of diminishing of ROS accumulation [5]. In *Arabidopsis thaliana*, both MYB12 and MYB75 independently promote the production of flavonoids. Overexpression of either gene or co-expression of both leads to flavonoid accumulation and reduced ROS levels in plants under drought stress [6]. Overexpression of UGT79B2/B3 greatly increases anthocyanin (a subclass of flavonoids) accumulation and improves antioxidant activity in response to drought [7]. Additionally, it has been proposed that the flavonoid synthesis pathway may be regulated by abscisic acid (ABA) and auxin (IAA) [8,9].

The NAC transcription factor (TF), consisting of NAM (No Apical Meristem), ATAF1 (*Arabidopsis thaliana* Activation Factor1), and CUC2 (Cup-shaped Cotyledon2), is a substantial family with a strongly conserved N-terminal domain and a variable C-terminal domain [10]. Numerous NACs have been discovered and reported to have various biological functions in plants. One CUC from *Arabidopsis* [11] and one NAM from Petunia [12] were found to regulate the formation of boundary cells in the meristem. Additionally, *AtNAC1* was observed to promote lateral root development [13], suggesting the involvement of NACs in tissue formation and development. Furthermore, NACs modulate various biological processes in plants. *SlNAC6* speeds up tomato fruit ripening [14]. Moreover, *BnaNAC60* regulates cell death, the accumulation of ROS, and leaf senescence in oilseed ripe protoplasts of tobacco [15], while *ONAC127* and *ONAC129* of *Oryza sativa* are linked to its grain filling [16]. Additionally, NACs are involved in the synthesis of plant secondary wall components. Four *PdeNAC* genes participate in the biosynthesis of the cellulose, galactoglucomannan, xylan, and lignin. Overexpression of the four *PdeNAC* genes in both tobacco and *Arabidopsis* leaves induces the deposition of secondary cell walls resembling xylem vessel cells [17].

*ANAC078 (also NAC2)*, an NAC transcription factor in Arabidopsis, regulates flavonoid biosynthesis [18]. And NAC is also involved in the regulation of color changes during fruit ripening [19]. For example, in peach (*Prunus persica*), one NAC was highly upregulated in blood-fleshed peaches and affected the biosynthesis of anthocyanin pigmentation [20]. Similarly, *MdNAC42* in apple (*Malus domestica* Borkh.) positively correlates with anthocyanin content as fruits ripened, and its overexpression in apple calli led to the upregulation flavonoid pathway genes, such as *MdCHS*, and *MdCHI* [21]. In addition to that, Apple calli overexpressing *MdNAC52* also accumulate anthocyanin [22]. Nevertheless, overexpressing *PaNAC03* led to aberrant embryo development in *Norway spruce* lines, and the three key flavonoid pathway genes were consistently downregulated [23]. However, there are no reports on the regulation of flavonoid biosynthesis by NAC transcription factors in leguminous plants.

During responses to water deficit, *SlNAC2*-overexpressing tobacco plants exposed to drought maintain a higher level of relative water content (RWC) and chlorophyll, increase proline levels, delay leaf senescence, and improve survival rates [24]. Overexpression of *SlJUB1* (which belongs to NAC) in tomato [25] and *VvNAC17* transgenic *Arabidopsis* has also been reported to enhance plant drought tolerance [26]. In rice, overexpression of *ONAC066* reduces water loss and the accumulation of ROS. Additionally, it increases the level of proline and soluble sugars [27]. *LpNAC13-*overexpressing tobacco plants exhibit an increase in antioxidant enzyme activity, proline, and chlorophyll contents and a decrease in MDA content [28]. Overexpression of OoNAC72 in *Arabidopsis* enhances the expression levels of stress-responsive genes [29]. These findings demonstrate that NACs play a positive role in regulating plant responses to drought. In addition to that, among plant hormones used to combat drought, dehydration promotes the production of abscisic acid (ABA), which subsequently controls plant responses to the stress [30]. Specially, ABA triggers a downstream signaling pathway. Therefore, delving into genes located in the ABA-dependent pathway downstream is crucial for illustrating the ABA-dependent mechanism of drought resistance in plants. However, it is necessary to investigate the relationship between phytohormones and plant resistance to drought, as well as the potential role of NAC transcription in mediating this process.

Several works on *NAC2* have been reported. In cowpea (*Vigna unguiculata* (L.) Walp.), overexpression of *VuNAC2* enhanced vegetative growth and provided tolerance to various abiotic stresses, including drought in a commercial cowpea cultivar. However, temporary suppression of *VuNAC2* led to significant severe growth inhibition [31]. The level of tanshinone IIA in *Salvia miltiorrhiza*, a flavonoid that has antioxidant activity, decreased after drought treatment [32]. In the same plant, overexpressing *SmNAC2* decreased the levels of tanshinone. Within the tanshinone biosynthetic pathways, *SmNAC2* overexpression downregulated CYP76AH1, whereas silencing it upregulated the *HMGR1*, *DXS2*, *KSL2*, and *CYP76AH1* expression levels [33]. However, there are missing linkages between NAC2 and vegetative growth or enhanced drought tolerance.

As a high-quality forage plant, *T. repens* is vital to agricultural advancement, offering a rich source of protein and minerals for a range of livestock. Simultaneously, *T. repens* roots can fix nitrogen, enhancing soil fertility and positively impacting soil improvement. In spite of these benefits, its growth and development are notably sensitive to environmental influences. For example, drought stress can easily affect *T. repens’* growth. The research on drought resistance genes in white clover indicates that the *TrSAMDC1* gene [34], *TrIAA18* gene [35], and *TrMYB33-TrSAMS1* module [36] present drought resistance capabilities. In addition, there are a few reports on stress-related NAC in white clover [37]. In our earlier study, we identified a NAC gene from drought-resistant white clover using RNA-seq and labeled it *TrNAC002*. Here, we transfer it into *Arabidopsis* and suppress its expression in white clover via the pTRV-VIGS system to reveal its initial biological functions in plants.

## 2. Results

### 2.1. Amplification, Identification and Analysis of TrNAC002

The amplification of *TrNAC002*’s CDS was successfully achieved (Figure S1A) using TrNAC002-F and TrNAC002-R (listed in Table S1). The sequencing results revealed that the CDS consists of 882 nt and encodes 293 amino acids (Figure S1B). The presence of one NAM domain in TrNAC002 was confirmed by the Pfam program (Figure S1C,D) and reconfirmed by Prosite (Figure S1E). According to phylogenetic analyses, TrNAC002’s closest counterpart is *Arabidopsis thaliana*’s AtNAC002, resulting in the gene being named TrNAC002 (Figure S2).

The theoretical isoelectric point (pI) of TrNAC002 is 5.77, and its molecular weight (MW) is 34.07 kDa. TrNAC002 has 43 negatively charged residues (Asp + Glu) and 38 positively charged residues (Arg + Lys). Its grand average of hydropathicity (GRAVY) is −0.895, indicating that the protein is hydrophilic. TMHMM analysis showed that TrNAC002 lacks a transmembrane structure. SignaIp5.1 predicted the absence of a signal sequence in TrNAC002, indicating that it is not a secretory protein. These results provide useful information for further research on TrNAC002. Netphosp 3.1 predicted 15 phosphorylation sites in TrNAC002, including serine, tyrosine, and threonine.

According to Weblogo’s analysis (Figure S3), the N-terminal conserved region of NAC transcription factors can be subdivided into five domains (I, II, III, IV, V; Figure S3). Subdomain I participates in functional dimer formation, while Subdomains III and IV are regions for DNA binding, which are positively charged and highly conserved and contain nuclear localization signals. Subdomains II and V are related to the functional diversity of NAC genes [38]. Simultaneously, certain amino acid sites were found to be highly conserved, such as leucine (Leu 14, Leu 38, Leu 83), proline (Pro 5, Pro 35), phenylalanine (Phe 3, Phe 44, Phe 45), glutamic acid (Glu 9, Glu 99), tryptophan (Try 63, Try 95), and glycine (Gly 61, Gly 67, Gly 78).

### 2.2. TrNAC002 Position on Chromosome and Promoter Analysis

According to the aligning results between the CDS sequence of *TrNAC002 and* the genome of white clover (see Figure S4A), the CDS can be divided into three segments that match with the corresponding regions located on chromosome 3P of white clover. To be more specific, 1 to 160 nucleotides of the CDS aligned with 46,192,871 to 46,193,030 nucleotides of chromosome 3P, 161 to 435 nucleotides aligned with 46,193,117 to 46,193,391 nucleotides, and 436 to 882 nucleotides aligned with 46,193,499 to 46,193,945 nucleotides. Thus, it was inferred that the *TrNAC002* gene comprises three exons and two introns. Figure S4B demonstrates its structure distribution model. Furthermore, there was a corresponding section on chromosome 5O of white clover, but it consisted of 21 gaps and some transitions and transversions, making it less ideal than the alignment result on chromosome 3P.

The PlantCARE result for the *TrNAC002* promoter region showed that 14 CAAT boxes (common cis-acting elements in promoter and enhancer regions) were located on the plus strand, nine ABREs (ABA-responsive elements), including one ABRE3a and one ABRE4, were positioned on the plus or minus strand, and one DRE (dehydration-responsive element) core were located on the plus strand.

### 2.3. TrNAC002 Is Located in the Nucleus and Exhibits Transcriptional Activation Activity

**Figure 1:**
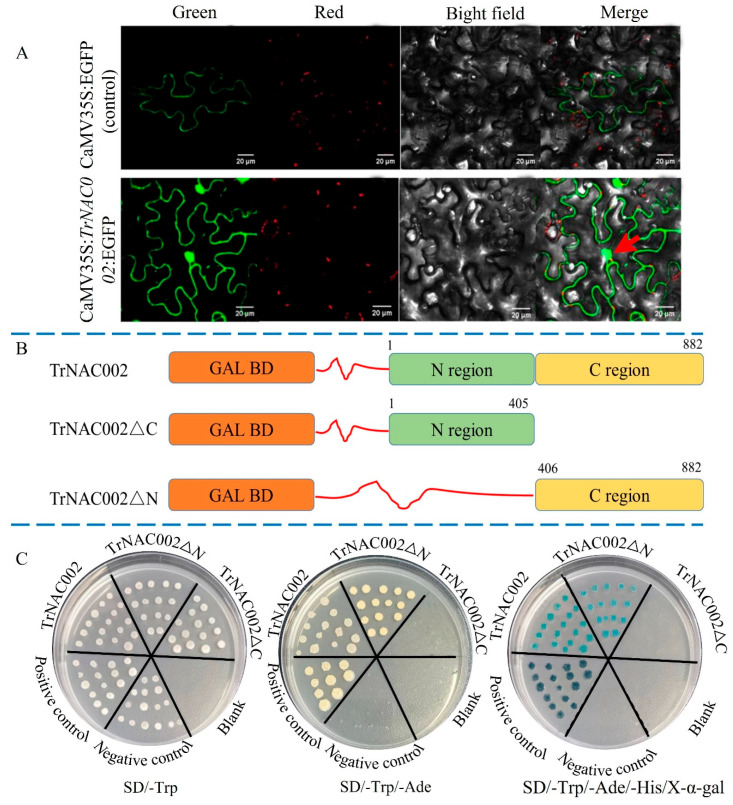
Subcellular localization and transcriptional activity assays of TrNAC002. (**A**): Subcellular localization of TrNAC002 based on visualization of EGFP. Red arrow indicates TrNAC002’s location in the nucleus. The fusion construct (CaMV35S:TrNAC002-EGFP) or empty vector (CaMV35S:EGFP, positive control) was transformed into the tobacco (*Nicotiana benthamiana*) leaves. Confocal microscopic images of epidermal cells were taken under green (for EGFP), red (for chloroplast fluorescence), and bright field. The images on the right were merged from those on the left. The scale bars represent 20 μm. (**B**): Schematic diagrams for constructing vectors used for transcriptional activity assays. Full-length and truncated fragments of TrNAC002 were introduced downstream of the GAL4-BD (galactose-specific transcription enhancing factor 4 binding domain) in the pGBKT7 vector. TrNAC002ΔC and TrNAC002ΔN indicate deletion of the C and N terminus, respectively. The numbers above the CDS represent the position of amino acid residues. (**C**): Growth of yeast (*Saccharomyces cerevisiae*) cells strain AH109 transformed with negative control (pGBKT7), positive control (pGBKT7-53 +pGADT7-T), and the three construct vectors on synthetic dextrose (SD)/-Trp, SD/-Trp/-Ade, and SD/-Trp/-Ade/-His plus X-α-gal (100 mg/L).

Cell-PLoc 2.0 and BUSCA predictions showed nuclear localization for TrNAC002. The fluorescent signals analysis of the enhanced green fluorescent protein (EGFP) and the TrNAC002-EGFP fusion protein in tobacco cells confirmed the nuclear localization of TrNAC002 (Figure 1A, indicated by red arrow), indicating its potential function as a transcription factor. To test the transcription-activating capacity of TrNAC002, we conducted a transactivation reporter assay using three constructed vectors (see Figure 1B). AH109 yeast cells transformed with pGBKT7 grew successfully on the SD/-Trp medium. However, only those transformed with constructs containing the full-length and the C region, alongside the positive control, displayed normal growth on the SD/-Trp/-Ade and SD/-Trp/-Ade/-His/X-α-gal medium, and demonstrated α-galactosidase activity (see Figure 1C). These results indicate that TrNAC002 has transcription-activating activity, with the activation domain located in the C region. Taken together, these results suggest that TrNAC002 could function as a transcription factor.

### 2.4. Expression Patterns of TrNAC002 in White Clover After Drought and ABA Treatment

**Figure 2:**
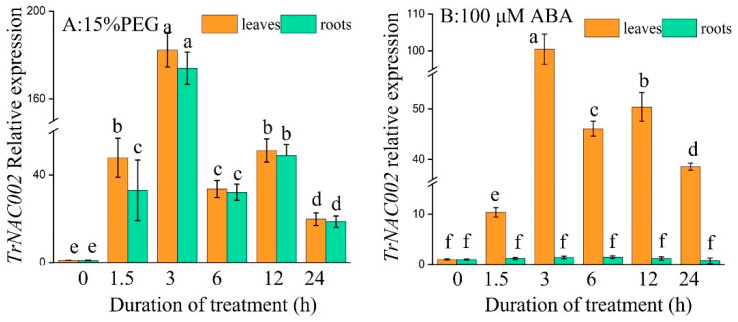
Expression patterns of *TrNAC002* in leaves and roots of white clover under 15% PEG and 100 μM ABA. (**A**): 15% PEG6000. (**B**): 100 μM ABA. Least Significant Difference (LSD) was used following ANOVA. Error bars represent the standard deviation (SD) of three independent biological replicates. Different letters above bars indicate statistically significant difference at *p* < 0.05.

Under 15% PEG, the expressions of *TrNAC002* significantly increased in both roots and leaves. In detail, *TrNAC002* expression in roots and leaves presented similar changes under drought stress, as both increased by about 147 times and 155 times that of the control at 3 h, respectively (Figure 2A). After the application of 100 μM ABA, the expression of the TrNAC002 gene rapidly increased to the highest level at 3 h (about 100 times), and then gradually decreased (Figure 2B)., while no significant differences were observed in the roots. The results indicate that *TrNAC002* can respond to drought and ABA treatments.

### 2.5. TrNAC002 Induced Vegetative Growth and Upregulated the Expressions of Growth-Related Genes in Arabidopsis thaliana

In total, four DH 5α colonies carrying the recombinant vector of *TrNAC002*-pBI121 were validated via PCR and electrophoresis (Figure S5A). Next, the recombinant vector (TrNAC002-pBI121) was transferred to *A. thaliana* through inflorescence infection in order to generate TrNAC002-overexpressing plants. *Arabidopsis* OE1 and OE12, with the highest relative expression of *TrNAC002* (Figure S5B), were selected for conducting the subsequent experiments.

**Figure 3:**
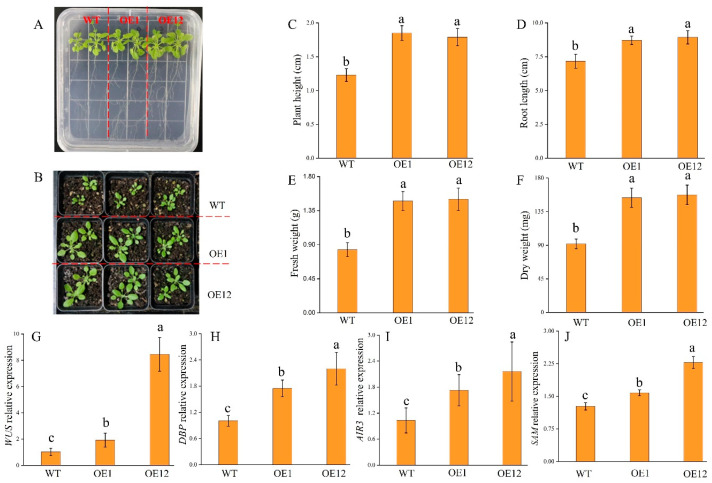
*TrNAC002* overexpression in *A. thaliana* stimulates its growth and expressions of genes associated with growth. (**A**,**B**): morphological appearances of OE plants and wild-type plants grow on 1/2 MS medium (**A**) and in growth chamber (**B**) for 15 days. (**C**): Plant height. (**D**): Root length. (**E**): Fresh weight. (**F**): Dry weight. (**G**): *WUS* relative expression. (**H**): *DBP* relative expression. (**I**): *AIR3* relative expression. (**J**): *SAM* relative expression. Least Significant Difference (LSD) was used following ANOVA. Error bars represent the standard deviation of three independent biological replicates. Different letters above bars indicate statistically significant difference at *p* < 0.05.

WT and *Arabidopsis* overexpressing (OE) seedlings were cultured separately on 1/2 MS medium (Figure 3A) and in a growth chamber for 15 days (Figure 3B). The plant appearance revealed that the OE plants grew at a faster rate than the WT. As predicted, *TrNAC002* enhances plant vegetative growth rate and biomass buildup in *Arabidopsis*. And the results show that plant height, root length, fresh weight and dry weight were significantly higher in OE plants than in WT (Figure 3C–F). The *SAM* and *WUS* genes participated in meristem formation [39], while the *DBP* gene was associated with lateral root formation, and the *AIR3* gene was linked to root cultures [1]. Thus, the relative expression levels of these genes was measured to reveal the regulatory mechanism of lateral root growth indirectly. The results indicate that the expression of these genes promoted the vegetative growth of *A. thaliana* (Figure 3G–J).

### 2.6. TrNAC002 Increased Drought Tolerance and Flavonoid Biosynthesis in A. thaliana

**Figure 4:**
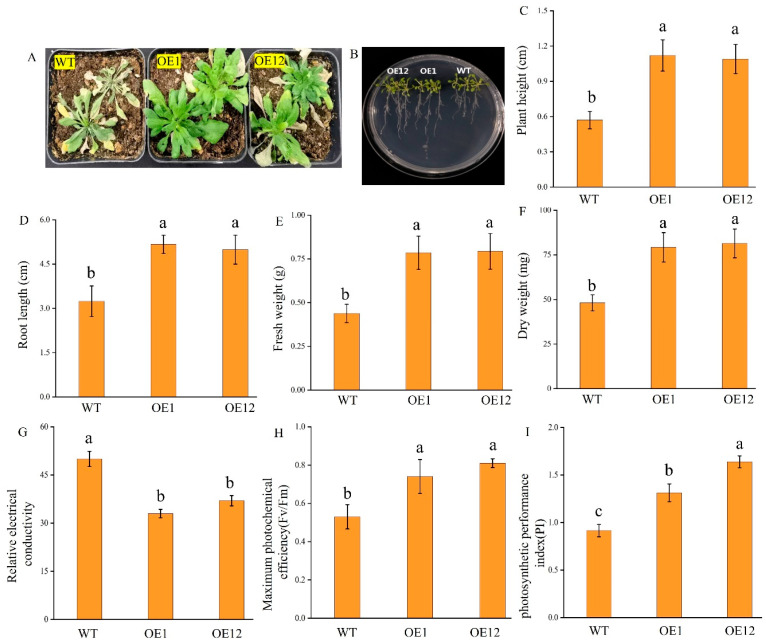
Phenotypic result of OE *Arabidopsis* and physiological comparison of OE and WT *A. thaliana* under drought stress. (**A**): natural drought, 12 days. (**B**): 200 mM mannitol in 1/2 MS medium, 7 days. For bar charts in Figure 4C–I, the plants were put under drought stress 7 days, and mannitol-induced drought stress was applied. (**C**): Plant height. (**D**): Root length. (**E**): Fresh weight. (**F**): Dry weight. (**G**): Relative electrical conductivity. (**H**): Maximum photochemical efficiency (Fv/Fm). (**I**): Photosynthetic performance index (PI). Least Significant Difference (LSD) was used following ANOVA. Error bars represent the standard deviation of three independent biological replicates. Different letters above bars indicate statistically significant difference at *p* < 0.05.

Briefly, 30-day-old wild-type (WT) and OE *Arabidopsis* plants were exposed to natural drought conditions for 12 days. The OE plants thrived in the arid soil, and most of their leaves retained their green color, while the WT plants were unable to retain water and wilted (Figure 4A). To investigate the differential response of the root systems of WT and OE *Arabidopsis* to drought stress, both were cultured on the 1/2 MS medium for 7 days and then transferred to medium containing 200 mM mannitol. The results show that the roots of OE plants were significantly longer than those of WT (Figure 4B). The results show that the plant height, root length, fresh weight and dry weight of OE plants were significantly higher than those of WT (Figure 4C–F). Meanwhile, the measurements of relative electrical conductivity, maximum photochemical efficiency (Fv/Fm), and photosynthetic performance index (PI) indicated that the overexpressing plants had better drought tolerance (Figure 4G–I).

**Figure 5:**
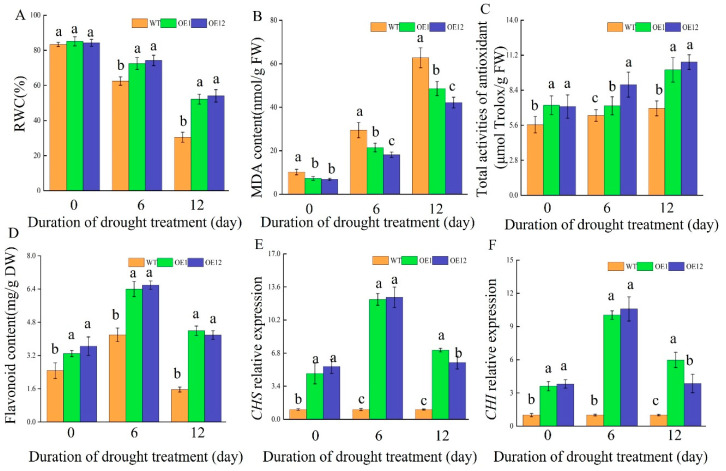

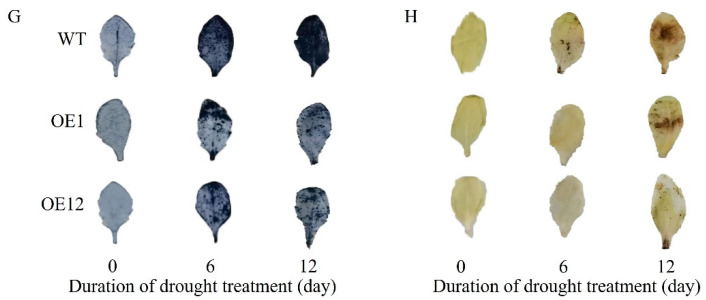
Physiological comparison of OE and WT *A. thaliana* under drought stress. (**A**): RWC under drought stress. (**B**): Malondialdehyde (MDA) content under drought stress. (**C**): Total activities of antioxidant after the treatment of drought. (**D**): Flavonoid content under drought stress. (**E**): Chalcone synthetase (CHS) relative expression. (**F**): Chalcone isomerase (CHI) relative expression. (**G**): accumulation of superoxide anion ($O_{2}^{-}$) detected by 3,3-diaminobenzidine (DAB) staining in leaves. (**H**): H_2_O_2_ accumulation determined after nitrobluetetrazolium (NBT) staining. Least Significant Difference (LSD) was used following ANOVA. Different letters above bars indicate a statistically significant difference at *p* < 0.05. Error bars depict SD of three independent biological replicates (**A**–**F**).

The results indicate that RWC in OE plants was significantly higher than in WT plants, suggesting that the overexpression of *TrNAC002* in *Arabidopsis* resulted in increased water availability (Figure 5A). We measured the MDA content and total antioxidant activities of OE and WT plants under natural drought stress. The results show that the MDA content level in WT plants was significantly higher than that in OE plants (Figure 5B), while OE plants had a higher level of total antioxidant activities than WT plants (Figure 5C). These findings indicate lower lipid peroxidation levels and an improved antioxidant capacity in transgenic plants. The flavonoid content of *Arabidopsis* with overexpression of *TrNAC002* under drought is displayed in Figure 5D. On the 6th day of drought stress, the flavonoid content of WT and OE plants reached its maximum. At each sampling time, flavonoid content in OE plants was significantly higher than that in WT plants, indicating that the overexpression of *TrNAC002* in *Arabidopsis* upregulated the biosynthesis of flavonoids. Consistent with the increase in flavonoid content, the expression levels of the two major enzymes in flavonoid synthesis, chalcone synthase (CHS) and chalcone isomerase (CHI), also increased significantly (Figure 5E,F). We then used NBT and DAB as chromogenic reagents to histochemically determine $O_{2}^{-}$ (Figure 5G) and H_2_O_2_ (Figure 5H), respectively. The results indicate that under drought stress, mutants overexpressing TrNAC002 have much lower ROS, visually, when compared to the control group.

### 2.7. Drought and ABA Induced TrNAC002 Expression and Flavonoid Accumulation in White Clover

**Figure 6:**
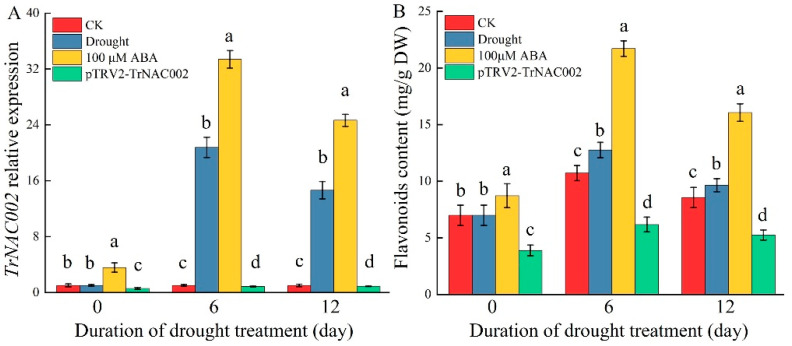

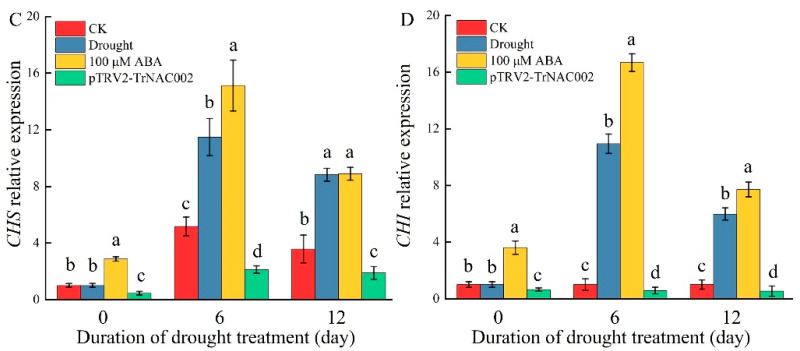
Expression of TrNAC002 and flavonoid content in white clover. CK, plants carrying empty pTRV2, served as a control group. (**A**): *TrNAC002* relative expression; (**B**): flavonoid content; (**C**) *CHS* relative expression; (**D**): *CHI* relative expression. Least Significant Difference (LSD) was used following ANOVA. Error bars represent the standard deviation of three independent biological replicates. Different letters above bars indicate statistically significant difference at *p* < 0.05. The pTRV-VIGS system resulted in negative symptoms in white clover.

In white clover, the relative expression of *TrNAC002* in leaves increased by approximately 20.78 times and 14.64 times under drought on the 6th and 12th days, respectively. Upon ABA treatment, *TrNAC002*’s relative expression increased by around 33.39 times and 24.65 times on the 6th and 12th days, respectively. Nevertheless, *TrNAC002*’s relative expression significantly declined after pTRV2-TrNAC002 treatment (Figure 6A). The content of flavonoids (Figure 6B), relative expression of *CHS* (Figure 6C), and relative expression of *CHI* (Figure 6D) exhibited similar change patterns with TrNAC002 expression, suggesting that both the application of 100 μM ABA and drought stress stimulated the expression of *TrNAC002* and the accumulation of flavonoids. The observed significant reduction in flavonoid content in pTRV2-TrNAC002 white clover suggests that TrNAC002 is a key regulator of flavonoid synthesis.

**Figure 7:**
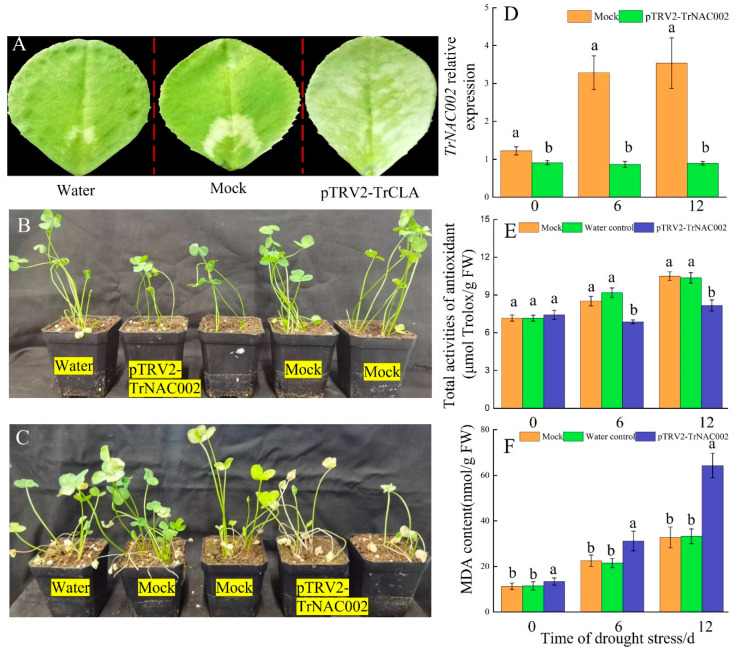
pTRV-VIGS and the TrCLA silencing in *T. repens*. (**A**): photobleaching phenotype was observed in pTRV1:pTRV2-TrCLA infected *T. repens* leaf. (**B**,**C**): morphological appearances of white clover in Mock and Water controls and pTRV2-TrNAC002 under normal condition (**B**) and natural drought for 6 days (**C**). (**D**): *TrNAC002* relative expression. (**E**): Total activities of antioxidant after the treatment of drought. (**F**): MDA content of white clover. Least Significant Difference (LSD) was used following ANOVA. Different letters above bars indicate a statistically significant difference at *p* < 0.05. Error bars depict SD of three biological replicates of data (**D**,**E**).

A recombinant containing one 306 nt *TrCLA* fragment regulated by a 2 × 35S promoter was constructed in order to access the effects of the pTRV-VIGS system on fresh white clover leaves. CLA is involved in chloroplast development and mutant plants with a CLA deficiency exhibit photobleaching in their leaves. The photobleaching phenotype was observed in pTRV2-TrCLA-infected seedlings, but no mosaic symptoms emerged in either the Mock (pTRV2) or the Water control (Figure 7A). After 30 days, white clover in the Mock and Water control groups exhibited better growth than those in the pTRV2-TrNAC002 group (Figure 7B), indicating that TrNAC002 is involved in white clover growth. The drought experiment revealed that white clover in the pTRV2-TrNAC002 group showed less tolerance to natural drought than in the Mock and Water control groups (Figure 7C), suggesting that the absence or reduction in TrNAC002 significantly compromises white clover’s drought resistance.

In addition, *TrNAC002*’s relative expression is displayed in Figure 7D, indicating that pTRV2-TrNAC002 treatment decreased the expression of *TrNAC002*. We also measured the MDA content and total antioxidant activities of Mock, Water control, and pTRV2-TrNAC002 white clover plants during natural drought stress. The results indicate that pTRV2-TrNAC002 plants had a reduced level of total antioxidant activity compared to the Mock and Water controls (Figure 7E). Furthermore, the MDA content level in pTRV2-TrNAC002 plants was significantly higher than that in the Mock and Water controls (Figure 7F), suggesting an elevated level of lipid peroxidation level in pTRV2-TrNAC002 plants and a diminished antioxidant capacity.

## 3. Discussion

### 3.1. TrNAC002 Duplication on Chromosome of White Clover

Allotetraploid genomics of white clover derived from *T. pallescens* as the maternal progenitor and *T. occidental* as the paternal progenitor [40]. The genome-blasting result showed that two *TrNAC002* genes appeared in the half-genome of white clover, one inherited from *T. pallescens* (Chromosome 3P), and another from *T. occidental* (Chromosome 5O). In plant-genomic architecture, gene and genome duplication is a prominent feature. Many researchers have continuously made an effort to determine the function of duplication in the evolution of the plant. Here, we found that *TrNAC002* failed to duplicate in the evolvements of *T. repens*, *T. pallescens*, and *T. occidental*, meaning that *TrNAC002* is a single-copy nuclear gene (scnDNA) in *T. repens* and its two progenitors.

Single-copy genes are commonly considered the most reliable molecular markers for inferring relationships between unresolved lineages [41]. Due to their uniqueness and high sequence conservation, they can be easily amplified and sequenced. Furthermore, scnDNAs differ from organelle genes, which are mostly inherited uniparentally and possess biparental inheritance. Therefore, scnDNAs could be effectively employed for managing speciation, hybridization, and incomplete lineage sorting of closely related plant species [42]. Furthermore, the use of multiple unlinked scnDNAs is more likely to reveal accurate relationships between species and resolve discrepancies among organelle genes. *TrNAC002* is a potential candidate for speciation and hybridization among white clover plants, as it is one of the scnDNAs. This contributes to the deduction of sub-species across white clover plants. Moreover, most of the scnDNAs that were identified belonged to housekeeping genes, which are constitutively expressed in organisms. Therefore, *TrNAC002* would play a crucial role in the biological pathway of white clover. The housekeeping gene’s function contributes to explaining TrNAC002’s visualization in the nucleus.

### 3.2. Expression Patterns of TrNAC002 in White Clover

In the current study, the expression of *TrNAC002* was significantly upregulated by drought stress in the leaves and/or roots of white clover (Figure 2A). TrNAC002 had higher expression levels in leaves when compared to its expression levels in roots at all sampling time points, indicating that TrNAC002 had a positive response to drought stress. In pearl millet, 151 NAC transcription factor genes were analyzed by researchers, resulting in 42 genes being affected by drought. After examining the expressions of 36 randomly selected NACs, Dudhate [43] found that many displayed drought responses in both roots and leaves. *Dactylis glomerata* L. exhibited high tolerance to various stresses, with 12 out of 108 NAC genes identified as responsive to drought stress [44]. Additionally, *CaNAC46* expression in *Capsicum annuum* was induced by drought and abscisic acid. The overexpression of *CaNAC46* in *A. thaliana* enhanced plants’ drought tolerance and increased lateral root growth [45]. The induction of *GhNAC4* expression in cotton by abiotic stresses and ABA led to the plant’s improved resistance to salinity and drought [46]. In our experiments, the application of ABA from an outside source enhanced the expression level of *TrNAC002* in leaves. This offers evidence that *TrNAC002* may have a prominent role in the positive response of white clover to drought stress.

In plants, ABA regulates their responses to dehydration and optimizes water use efficiency. Local drought signals stimulate ABA production in various plant organs, with a more efficient process in leaves than in roots [47]. Accumulated ABA then activates the downstream signaling pathways. Overexpression of UGT79B2/B3 and performs its function with feedback from other paths [48]. It was reported that ABA increased under salt stress [49], cold stress [50], and heat stress [51]. The *TrNAC002* promoter contained nine ABRE motifs involved in abscisic acid responsiveness, which were located on both the plus and minus strands, which largely explains the high level of *TrNAC002* expression under abiotic stress and ABA treatment.

### 3.3. TrNAC002 Overexpression in Arabidopsis Conferred Abiotic Resistance and Influenced Expression Profiles Related to Growth

After planting *Arabidopsis* in square Petri dishes for 20 days, OE plants had larger leaves and longer roots than WT plants. It is well known that the root system can adjust the overall architecture of the root system to respond to changes in soil moisture. When water was scarce, the root system reconfigured its architecture to improve its ability to absorb water. In our experiments, the OE plants with longer taproots and more lateral roots may have a higher survival rate under drought stress, indicating that *TrNAC002* would have potential application value in plant drought research. NACs in plants had different effects on drought stress. In wheat (*Triticum aestivum* L.), transgenic plants overexpressing *TaSNAC4-3A* reduced stomatal aperture size to reduce water loss rate [52]. In tomato, the overexpression of *SlNAC6* resulted in a significant delay in growth accompanied by lower water loss and improved tolerance to PEG stress [14].

NACs regulate the expression of growth-related genes. *PwNAC1* mediates drought stress in *Picea wilsonii* by significantly increasing under drought and activating expression of *ERD1* gene [53]. In tomato (*Solanum lycopersicum*), the NAC factor JUNGBRUNNEN1 (JUB1) controls the expression levels of *SlDREB1*, *SlDREB2*, and *SlDELLA* by directly binding to their promoters [25]. In rice, the NAC transcription factor *ONAC066* binds to a cis-element of the *OsDREB2A* promoter, promoting the transcription of *OsDREB2A* [27]. In this study, *TrNAC002* also increased the expression of genes associated with root growth. As is well known, root growth results from the coordination of cellular processes, including division, elongation, and differentiation in the root apex. The *WUS* gene serves as a key regulator for maintaining shoot meristem integrity in *A. thaliana* by encoding the homeodomain TF [54]. The overexpression of *WUS* also governs shoot formation and development [55] and contributes to the growth of both taproot and lateral root due to increased expression levels.

Both the *DBP* and *AIR3* genes were found to be auxin-responsive and play a crucial role in root formation. The *DBP* gene encodes a DNA-binding protein, which is universally expressed in plants. *TrNAC002* was seen to upregulate its expression. Its expression level increased by fourfold in tissues with rapid cell division [56]. *AIR3* encodes subtilisin-like proteases, and its expression level increases in the roots after NAA treatment [57]. Our findings showed that DBP and AIR3 expression levels increased in *TrNAC002*-OE12 *Arabidopsis*. Additionally, the results demonstrated that the overexpression of *TrNAC002* led to enhanced lateral root formation, which strongly suggests TrNAC002’s involvement in the auxin signal during the root developmental process.

The shoot apical meristem (*SAM*) gene is associated with the aboveground portion of plants. The shoot apex sustains continuous aboveground organogenesis and embryogenesis establishment is correlated with the *SAM*. In *Arabidopsis*, the *SAM* interacts with AMs (Axillary meristems) to generate aboveground portions of the plant and to determine plant architecture. In this study, the aboveground parts of *Arabidopsis* OE plants were larger than those of WT plants, suggesting an active role of *TrNAC002* in improving the aboveground parts.

### 3.4. TrNAC002 Upregulated Flavonoid Accumulation and Enhanced Plant Drought Resistance

The overexpression of *TrNAC002* in *A. thaliana* significantly increased the total activities of antioxidants, resulting in a reduction in the accumulation of superoxide anion and hydrogen peroxide, which, in turn, decreased the production of malondialdehyde. These changes improved the growth conditions of the plant, making it more resilient to drought stress. Interestingly, metabolite detection results showed a remarkable increase in the content of flavonoids. Flavonoids can be induced by drought stress and their accumulation may trigger feedback regulation of plant stress tolerance [1]. To date, research on the synthesis of drought-induced flavonoids has made substantial progress in elucidating physiological phenotypes and molecular mechanisms. In the present study, *TrNAC002* was significantly induced by drought stress, and *Arabidopsis* plants with *TrNAC002* overexpression exhibited enhanced drought resistance and increased levels of flavonoids. Interestingly, *ANAC078* (also known as *NAC002*) in *A. thaliana* has been previously reported to regulate flavonoid biosynthesis [18]. Combined, these results suggest that *NAC002* was involved in regulating flavonoid synthesis.

In addition, ABA was also induced by drought and consequently could trigger the ABA signal transduction pathway. In this study, we treated white clover with exogenous ABA and observed an increase in both *TrNAC002* expression and flavonoid content. This suggests that ABA may regulate *TrNAC002* expression, thereby promoting flavonoid production. In the pTRV-VIGS experiment, the expression of *TrNAC002* decreased and, consequently, the reduction in flavonoid content was less pronounced. These results suggest that other transcription factors also play a role in regulating flavonoid synthesis.

**Figure 8:**
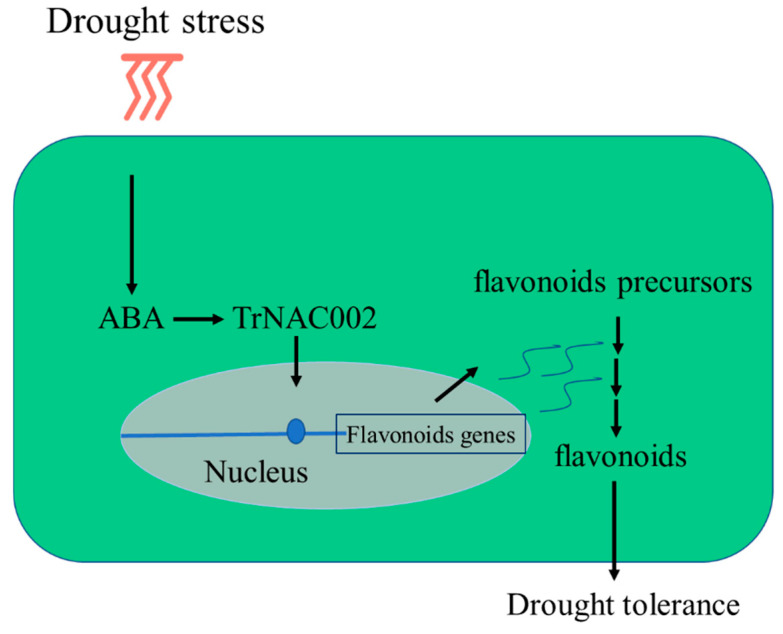
A simple proposed working model illustrating the regulatory role of abscisic acid (ABA)-responsive TrNAC002 in flavonoid accumulation under drought stress. Drought upregulates ABA biosynthetic genes and increases endogenous ABA, which subsequently triggers the ABA signaling transduction pathway, leading to synthesis of TrNAC002. As a result, TrNAC002 positively regulates the expression of flavonoid genes, which are then integrated into the metabolic pathway for promoting flavonoid biosynthesis. The flavonoid synthetic proteins are shown using the wavy lines.

Taken together, we propose a working model for understanding how TrNAC002 improves plant drought tolerance. Drought exposure increases endogenous ABA synthesis by upregulating ABA biosynthetic genes, which triggers the ABA signaling pathway to produce the TrNAC002 protein. Then, TrNAC002 upregulates the expression of flavonoid synthesis genes and increases flavonoid synthesis (see Figure 8). This model helps to uncover the molecular mechanisms and transcriptional network of flavonoid accumulation in response to drought. Additionally, our work provides a new insight into understanding the contribution of TrNAC002-orchestrated ABA signaling in modulating flavonoid synthesis. These findings from our study reveal a transcriptional module consisting of TrNAC002 that underlies ABA- and drought-induced flavonoid synthesis in plants.

## 4. Materials and Methods

### 4.1. Study Species

*T. repens* is a perennial plant. Its seeds can sprout rapidly when conditions are favorable and grow swiftly. The plants are usually low and the stems are weak. It is widely distributed and planted worldwide. In China, the distribution of white clover is mainly concentrated in the northern region. *T. repens* is a high-quality feed plant with high nutritional value. It is rich in protein and minerals, and suitable as livestock feed. In addition, *T. repens* also has a good effect on covering the ground and preventing soil erosion; so, it has also been widely used in agricultural production and ecological protection.

### 4.2. Growth Conditions for Plants

The seeds of *Nicotiana benthamiana* (cv. k326, purchased from HUAYUEYANG BIOTECHNOLOGY(BEIJING) Co., Ltd., Beijing, China) were selected and sown in plastic pots (8 cm in length and width, 15 cm in depth); the nutrient soil was a mixture of Pindstrup substrate and vermiculite with a ratio of 9:1 (*v*/*v*). The uniform-sized seeds of *T. repens* (cv. Ladino, purchased from Beijing Mammoth Seed Industry Company, Beijing, China) were planted in plastic boxes (30 cm in length, 25 cm in width, and 10 cm in depth) loaded with quartz sand in which the particle diameter was 0.3 to 0.5 cm, and cultured with Hoagland’s nutrient solution (HB8870-9, Qingdao Haibo Biotechnology Co., Qingdao, China). The culture conditions were 23 °C for 12 h in the day and 19 °C for 12 h in the dark, with approximately 220 μmol/(m^2^·s) of photosynthetically active radiation (PAR).

The ecotype Col-0 (purchased from HUAYUEYANG BIOTECHNOLOGY (BEIJING) Co., Ltd., Beijing, China) and *TrNAC002*-overexpressing *Arabidopsis* were used to evaluate the effects of drought stress. After sterilizing the *Arabidopsis* seeds with alcohol (75%, *w*/*v*, 3 min) and NaClO (1%, *w*/*v*, 8 min), we sowed them on 1/2 MS medium with 3% sucrose and 0.7% agar in Petri dishes and then cultured in the growth chamber, 21 °C for 10 h in the day and 19 °C in the dark for 14 h, with approximately 150 μmol/(m^2^·s) of photosynthetically active radiation (PAR). After 12 days, uniformly growing *Arabidopsis* seedlings were transplanted into plastic pots filled with nutrient soil (Pindstrup substrate and vermiculite with a ratio of 9:1, *v*/*v*).

### 4.3. Isolation and Analysis of TrNAC002 Gene of White Clover

Analysis of an earlier transcriptome dataset of white clover unraveled the mRNA sequence of *TrNAC002* (MT536937). The full length of *TrNAC002* was obtained using the SMARTer^®^ RACE 5′& 3′Kit (Clontech Laboratories, California, USA). After predicting the open reading frames (ORFs) of *TrNAC002* online (https://www.ncbi.nlm.nih.gov/orffinder (accessed on 17 October 2024)), we designed a pair of primers (TrNAC002-F and TrNAC002-R, Table S1) to acquire the complete coding sequence (CDS) of *TrNAC002*.

After acquiring the CDS of *TrNAC002*, we obtained its primary peptide sequence online (https://www.expasy.org/resources/translate (accessed on 12 October 2024)). Subsequently, we uploaded the CDS protein sequence to the UniProt database (https://www.uniprot.org/blast/ (accessed on 15 October 2024)) to perform BLAST. In addition, the HMM (hidden Markov model) profile of the NAC domain was confirmed through the Pfam database (http://pfam.xfam.org/ (accessed on 10 October 2024)) and the Prosite website (https://prosite.expasy.org/ (accessed on 17 October 2024)). Next, we analyzed the primary characteristics of TrNAC002 through PortParam (https://web.expasy.org/cgi-bin/protparam/protparam (accessed on 17 October 2024)) and calculated the grand average of hydropathicity (GRAVY) of it on ProtScale (https://web.expasy.org/protscale/ (accessed on 16 October 2024)).

TrNAC002 and *Arabidopsis* NAC proteins downloaded from PlantTFDB (http://planttfdb.gao-lab.org/family.php?fam=NAC (accessed on 10 October 2024)) were used to construct a phylogenetic tree via MEGA7 [58]. The amino acid frequency of the conserved region of NAC transcription factors was displayed through the default settings of Weblogo (http://weblogo.threeplusone.com/create.cgi (accessed on 11 October 2024)).

### 4.4. Subcellular Localization of TrNAC002 and Its Transcription Activity Assay

Cell-PLoc 2.0 (http://www.csbio.sjtu.edu.cn/bioinf/Cell-PLoc-2/ (accessed on 12 October 2024)) and BUSCA (http://busca.biocomp.unibo.it/ (accessed on 11 October 2024)) contributed to predicting subcellular localization. We also amplified the CDS of *TrNAC002* with the homologous primers TrNAC1132-SF and TrNAC1132-SR (listed in Table S1) (termination codon excised, primers designed online (https://www.takarabio.com/learning-centers/cloning/primer-design-and-other-tools (accessed on 15 October 2024)) and inserted it upstream of the enhanced green fluorescent protein (EGFP) of the pYBA-1132 (KF876796) vector. Then, the recombinant plasmids CaMV35S:TrNAC002: EGFP and CaMV35S:EGFP were separately transformed into 45-day-old *Nicotiana benthamiana* leaves. After two days, the GFP fluorescence was observed through a fluorescence microscope (Carl Zeiss710 SAS, Jena, Germany).

We inserted *TrNAC002* and two truncated fragments (N-terminal and C-terminal regions) into the pGBKT7 vector (Clontech Laboratories, California, USA.) to assess transcriptional activation activity. Yeast strain AH109 was independently transformed with the construct vectors and cultured on SD/-Trp, SD/-Trp/-Ade, or SD/-Trp/-Ade/-His/X-α-gal (20 mg/L). Concurrently, the pGBKT7 and pGBKT7-53 +pGADT7-T vectors were used as negative and positive controls, respectively.

### 4.5. Chromosome Positioning, Gene Duplication Analysis for TrNAC002

The BLAST GUI Wrapper program in the TBtools [59] was run by using *TrNAC002* sequences as the query and the white clover genome sequence downloaded from NCBI (https://www.ncbi.nlm.nih.gov/genome/13404 (accessed on 10 October 2024)) as the database to determine the position and duplication of *TrNAC002* in chromosome of white clover.

### 4.6. Analysis of TrNAC002 Promoter

Due to the incompleteness of the white clover genome, a sequence only 1664 nt in length ahead of the *TrNAC002* gene on Chromosome 3P was used as its promoter region, and promoter analysis was subsequently performed on the website PlantCARE (http://bioinformatics.psb.ugent.be/webtools/plantcare/html/ (accessed on 10 October 2024)).

### 4.7. Production of Arabidopsis thaliana Overexpressing TrNAC002

*TrNAC002* CDS without the termination codon was amplified (using TrNAC121SF and TrNAC121SR primers listed in Table S1) and cloned to the downstream of the CaMV35S in a pBI121 vector with the Ready-to-Use Seamless Cloning Kit (Sangon Biotech, Shanghai, China). CaMV35S:TrNAC002 was transferred into *A. thaliana* via the *Agrobacterium tumefaciens* (EHA105)-floral dipping method [60]. We planted the obtained seeds in 1/2 MS medium containing 100 mg/L kanamycin, and the seedlings with normal growth and greening of leaves were used as T_0_-generation-positive plants, and after normal culture of these positive plants, the DNA was extracted from the leaves and verified by PCR with the fragment (196-bp) of kanamycin resistance gene (NPT). The positive seedlings of *A. thaliana* of the T3 generation were used for the subsequent experiment. The seedlings of WT *A. thaliana* and OE *A. thaliana* were cultivated on 1/2 MS medium or in a growth chamber for 15 days.

### 4.8. Drought Stress and ABA Treatment in White Clover

Briefly, 30-day-old white clover plants were used to confirm the relative expression level of *TrNAC002* under natural drought and 100 μM ABA. For natural drought, these were thoroughly watered only at the beginning of the experiment and not again after the start of experiment. The length treatment time was 0, 6, and 12 d. The second mature leaves of plants and 3/4 of the roots were sampled for each plant and immediately stored at −80 °C for subsequent experiments.

### 4.9. qRT-PCR of TrNAC002 in White Clover and Expression Profiles Related to Growth in Arabidopsis

Briefly, 30-day-old white clover plants were immersed in 15%PEG and 100 μM ABA to detect the expressions of *TrNAC002* under drought for 24 h. The sampling times were 0 h, 1.5 h, 3 h, 6 h, 12 h and 24 h after the beginning of the experiment. Total RNA was isolated from root and leaf tissues through HiPure Universal RNA Kit. Then, 0.5 μg of total RNA was reverse-transcribed for cDNA synthesis through PrimeScript RT reagent Kit with gDNA Eraser. To examine the *TrNAC002*, *SAM*, *WUS*, *DBP*, and *AIR3* gene expression, quantitative real-time PCR was performed using a step-one real-time procedure and NovoStart SYBR qPCR Supermix Plus (Novoprotein, Suzhou, China). Reactions were carried out in triplicate for each reaction, and 10 μL of the total volume consisted of 1 µL of cDNA, 0.5 μL of each primer (10 μM/μL), and 5 μL of NovoStart SYBR qPCR Supermix Plus. The PCR procedures were as follows: initial denaturation at 95 °C for 1 min, 39 cycles of 95 °C for 5 s, 60 °C for 30 s, and 95 °C for 5 s, and melt-curve analysis. According to the result of our previous reference primer selection experiment for some genes, the β-Actin was used as a reference gene. Relative fold differences were calculated by using the 2^−∆∆CT^ method [61]. The qRT-PCR primers for *TrNAC002*, *SAM*, *WUS*, *DBP*, *AIR3*, *CHS*, and *CHI* gene are listed in Table S1.

### 4.10. Measurement of Physiological Parameters

After cultivation of WT *A. thaliana* and OE *A. thaliana* on 1/2 MS medium for 15 days in a growth chamber, plant height, root length, fresh weight and dry weight were measured.

Then, 30-day-old T_3_ homozygous lines of *TrNAC002* transgenic *Arabidopsis* were exposed to drought. After 12 days of drought treatment, we observed the phenotypes of the seedlings and conducted index measurement experiments.

For the relative water content (RWC), about 0.1 g of fresh leaves was recorded as fresh weight (FW). Then, the leaves were dipped into distilled water for 24 h at 4 °C to become saturated fresh weight (SW), followed by drying for 30 min at 105 °C and then drying for 48 h at 75 °C. After 3 d, the dry weight (DW) was recorded. The RWC was obtained by way of the following calculation: RWC(%) = (FW − DW)/(SW − DW) × 100%.

For relative electrolyte conductivity, ~0.1 g leaves were wrapped with tissue and soaked into ddH_2_O for 24 h in a 25 °C incubator; then, initial conductivity (S1) was conducted by using a conductivity meter. Subsequently, the samples were incubated in a water bath at 100 °C for 15 min and cooled down at room temperature to measure the second conductivity (S2). The final relative electrical conductivity was El = (S1/S2) × 100%.

After the fresh weight of the leaves of each plant line was weighed and dried at 75 °C to a constant weight, this was recorded as fresh weight and dry weight, respectively. The whole plant leaves were kept in the dark for 30 min, and then the maximum photochemical efficiency (Fv/FM) and photosynthetic performance index (PI) were measured by plant chlorophyll fluorescence efficiency instrument (Pocket PEA, Hansatech, English).

For visualizing H_2_O_2_ and $O_{2}^{-}$ accumulation in leaves, we used 1 mg/L 3,3-diaminobenzidine (DAB) and 0.5 mg/L nitrobluetetrazolium (NBT) for histochemical coloration, respectively. In the present study, we sampled three fresh leaves from the individual plant and immersed them in DAB and NBT solution at room temperature for 20 h, respectively. Then, the materials were fully decorated with absolute ethanol for 20 min, under the condition of a boiling water bath, according to a previous study [62].

Leaf tissues (0.1 g) were flash-frozen in liquid nitrogen, quick-ground on ice with 2 mL of cold PBS (50 mM, pH 7.8, 4 °C), and then centrifuged for 10 min (4 °C, 12,000 rpm). The supernatant was used for measuring the malondialdehyde (MDA) level and total activity of antioxidant assays. For MDA, 0.5 mL of the supernatant was mixed with 1 mL reaction solution containing thiobarbituric (0.6%, *w*/*v*) and trichloroacetic acid (10%, *w*/*v*). The absorbance of the reaction solution was detected at 532 and 600 nm. The total activity of the antioxidant of the supernatant was quantified using specific test kits (G0142W, Grace Biotechnology Co., Suzhou, China) through ABTS (2,2-Biazobis (3-ethylbenzothiazole-6-sulfonic acid) diammonium salt) assay, according to the manufacturer’s instructions.

The total content of flavonoids in leaves was measured through an ELISA test kit (UPLC-MS-4292, UPLC-MS, Shanghai Liquid Quality Testing Technology Co., Shanghai, China), and sampling, testing, and calculating were carried out according to the manufacturer’s instructions.

### 4.11. Virus-Induced Gene Silencing (VIGS) forTrNAC002 in White Clover

Vector pTRV1 (AF166084.1) and pTRV2 (AF406991) were used as the pTRV-VIGS vectors, and their detailed information was described in [63]. Total RNA was extracted from about 2-week-old young white clover leaves, and the first strand of cDNA was synthesized. The conserved 306 nt fragment of the *TrCLA* (CLA stands for the cloroplastos alterados gene, which encodes 1-deoxyxylulose-5-phosphate synthase. This gene is involved in chloroplast development and is highly conserved in evolution. Mutant plants with a CLA deficiency exhibit photobleaching in their leaves. Here, CLA mutant white clover is a positive control plant.) and 394 nt fragment of *TrNAC002* (MT536937) gene were obtained through PCR using synthesized cDNA as a template and inserted into the vector pTRV2 (Figure S6A). The primers for *TrCLA* were TrCLA-306-F and TrCLA-306-R, and the ones for *TrNAC002* were TrNAC-394-F and TrNAC-394-R. The fragments were amplified with homologous primers designed online (https://www.takarabio.com/learning-centers/cloning/primer-design-and-other-tools (accessed on 15 October 2024)). The extracted pTRV2 vector was digested with restriction endonuclease EcoRI and KpnI (TAKARA) and then purified with Gel Extraction Kit. Then, 3 μL pTRV2, 2 μL PCR product, and 5 μL Seamless Cloning Master mix (Ready-to-Use Seamless Cloning Kit, Sangon Biotech, Shanghai, China) were mixed and incubated at 50 °C for 20 min. Subsequently, 10 μL of the mixture was transformed into 100 μL *E. coli* DH5α competent cells. Transformants were cultured on an LB culture medium with 1% agar at 37 °C for over 16 h and tested by PCR amplification. The recombinant plasmid from the positive colony was purified and sequenced. The final selected plasmid was then transformed into GV3101 (*Agrobacterium tumefaciens* strain) to conduct the infection experiment of white clover, and the infection solution was a mixture of pTRV1/GV3101 and pTRV2/GV3101 with OD_600_ of 0.6 and a volume ratio of 1:1, as illustrated in Figure S6B.

After infestation, white clover seedlings were planted in nutrient soil for normal culture. Seedling leaf phenotypes were observed daily. Thirty days later, a natural drought experiment was conducted for 6 days to determine physiological indices. Here, white clover injected with water was a blank control, white clover injected with pTRV2 was a negative control (Mock), and white clover injected with pTRV2-TrCLA served as positive control.

### 4.12. Statistical Analysis

All the data were processed using Origin 2021. Student’s t test is used as a parametric test to compare two means. Statistical differences were analyzed through ANOVA based on Fisher’s least significant difference at the levels of *p* < 0.05.

## 5. Conclusions

The results of the analysis showed that *TrNAC002* is a single-copy nuclear gene in *T. repens* and its two progenitors, *T. pallescens* and *T. occidental*. This unique feature makes *TrNAC002* a valuable molecular marker for determining relationships between uncertain lineages. TrNAC002 is a member of the NAC family, as revealed by bioinformatic analysis. Our experiments demonstrate that TrNAC002 exhibited two distinct biological functions, promoting plant growth and enhancing plant resistance to drought via the modulation of flavonoid synthesis.

Moreover, analyzing the TrNAC002 transcription factor helps elucidate the molecular mechanisms of plant growth and development, providing a theoretical framework for genetic advancements in plants. By employing biotechnology, including gene editing and transgenic technology, the TrNAC002 transcription factor can be used to produce plant varieties with superior drought resistance, contributing to the sustainability of agriculture.

## Data Availability

The data will be available on request.

## Supplementary Materials

The following supporting information can be downloaded at: https://www.mdpi.com/article/10.3390/plants14010031/s1**,** Figure S1: Clone of TrNAC002 and its identification and annotations. A: PCR amplification of TrNAC002 gene. B: Amino acid sequence of TrNAC002 protein. C and D: NAM domain in TrNAC002. E: Reconfirming result of TrNAC002 by Prosite; Figure S2: A phylogenetic tree constructed based on TrNAC002 and NAC proteins of Arabidopsis thaliana, generated by neighbor-joining method. The colored shading highlights the relationship between TrNAC002 and AtNAC002 of Arabidopsis thaliana; Figure S3: Analysis for conserved motifs of NAC transcription factors; Figure S4: Analysis for Chromosomal distribution, gene structure illustration of TrNAC002. A: aligning result of TrNAC002 segments to chromosome 3P of white clover. B: illustration model of TrNAC002 structure; Figure S5: Recombinant plasmid identification(A), relative expression of TrNAC002 in TrNAC002 overexpressing Arabidopsis thaliana(B), and PCR identification of positive plants; Figure S6: Schematic representation of pTRV VIGS- system construction and TrCLA silencing in white clover. A: Genomic organization of the pTRV1 and pTRV2. Boxes indicate Open reading frames (ORF). The 306-bp fragment of TrCLA and the 394-bp fragment3 of *TrNAC002* are inserted in pTRV2. B: Illustration of infection experiment of white clover with mixture of pTRV1/GV3101 and pTRV2/GV3101; Table S1: List of primer sequences.

## Abbreviations

OE: overexpressing; WT: wild type; SAM: shoot apical meristem; WUS: WUSCHEL; DBP: DNA-binding protein; AIR3: auxin-induced in root cultures3; ROS: reactive oxygen species; CHS: chalcone synthase; CHI: chalcone isomerase; MDA: malondialdehyde; TF: transcription factor; ABA: abscisic acid; GFP: green fluorescent protein.
