# Supplementary material for: The White Clover Single-Copy Nuclear Gene TrNAC002 Promotes Growth and Confers Drought Resistance in Plants Through Flavonoid Synthesis
Source: Plants (Basel). 2024 Dec 25;14(1):31. doi: 10.3390/plants14010031 (PMC11722983; doi:10.3390/plants14010031)
Supplement: Supplementary file 1 [file plants-14-00031-s001.zip › plants-3293724-supplementary.pdf]

Supporting information

Table S1 List of primer sequences

Primer	Sequence
TrNAC002-F	5'-ATGCAGGGTGAATTAGAATTGCCAC-3'
TrNAC002-R	5'-TCAAAATGGTTTTTGTTGGAACATG-3'
β-actin-F	5'- CTCCTTCCATACTGGTCTCCTCCGC-3'
β-actin-R	5'- GCCCAAACATTAGGTGGTCTT-3'
TrNAC121-SF	5'-AGAACACGGGGGACTCTAGAATGCAGGGTGAATTAGAATTGC-3'
TrNAC121-SR	5'-TAAGGGACTGACCACCCGGGTCAAAATGGTTTTTGTTGGAAC-3'
TrNAC1132-SF	5'-GTGGATCCCCCGGGCTGCAGATGCAGGGTGAATTAGAAT-3'
TrNAC1132-SR	5'-CGGGCCCCCCCTCGAGTCAAAATGGTTTTTGTTGGAACA-3'
pBI121GUS-F	5'-TGGATCGCGAAAACTGTGGA-3'
pBI121GUS-R	5'-CGCAAGTCCGCATCTTCATG-3'
qNAC002-F	5'-CTAATCGGGCTGCTGGAAG-3'
qNAC002-R	5'-GCGCTTTACCGGCATAGAA-3'
qSAM-F	5'-CGCAAGTCCGCATCTTCATG-3'
qSAM-R	5'-CGCAAGTCCGCATCTTCATG-3'
qWUS-F	5'-CCAGCTTCAATAACGGGAA--3'
qWUS-R	5'-TCATGTAGCCATTAGAAGC-3'
qDBP-F	5'-TGACGGGGTTTGGGATG-3'
qDBP-R	5'-AAGTTCGGTGGTGGCTGTG-3'
qAIR3-F	5'-TCGCTGCTCATCTAGACCAC-3'
qAIR3-R	5'-CCATGATCGAGTCGTGTGAA-3'
TrCLA-306-F	5'-GTGAGTAAGGTTACCGAATTCGGCGTTACCAAGTTTGATCC-3'
TrCLA-306-R	5'-GAGACGCGTGAGCTCGGTACCATCATAGCCCCTCTGCAAG-3'
TrNAC-394-F	5'-GTGAGTAAGGTTACCGAATTCTCCAAGGGATCGGAAATACCC-3'
TrNAC-394-R	5'-GAGACGCGTGAGCTCGGTACCCAATTGGTAGTTCCCCAATTTC-3'

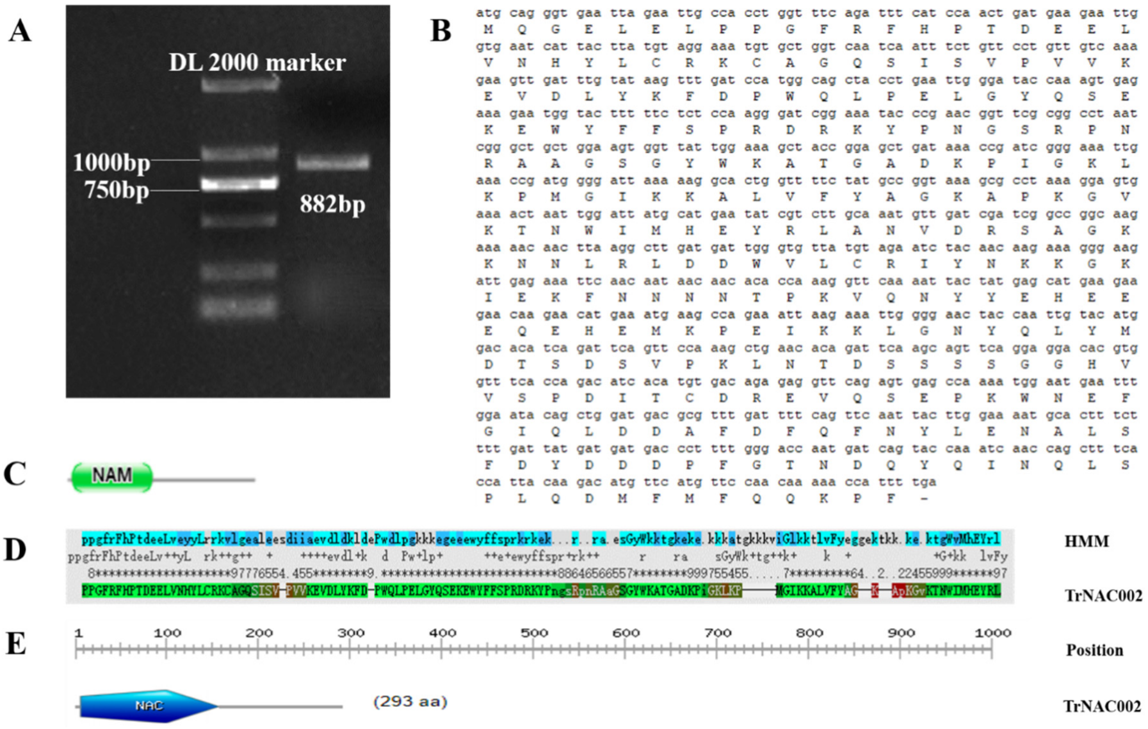

Figure. S1 Clone of *TrNAC002* and its identification and annotations. A: PCR amplification of *TrNAC002* gene. B: Amino acid sequence of TrNAC002 protein. C and D: NAM domain in TrNAC002. E: Reconfirming result of TrNAC002 by Prosite.

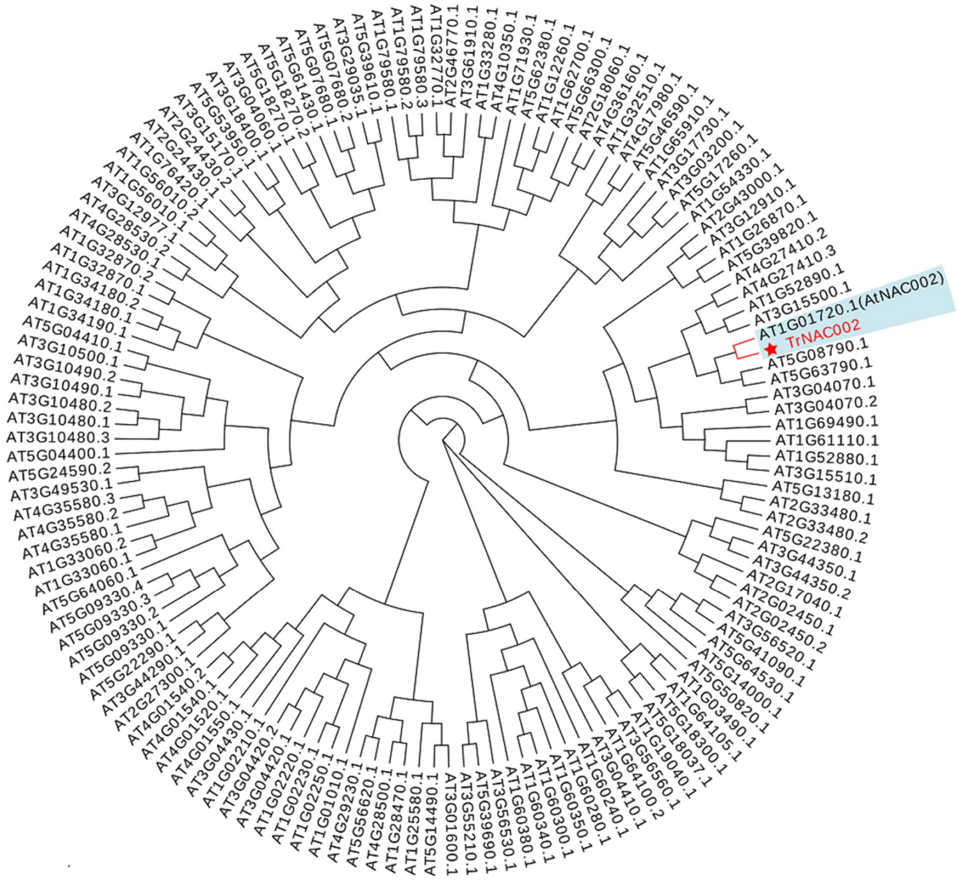

Figure.S2 A phylogenetic tree constructed based on TrNAC002 and NAC proteins of *Arabidopsis thaliana*, generated by neighbor-joining method. The colored shading highlights the relationship between TrNAC002 and AtNAC002 of *Arabidopsis thaliana*.

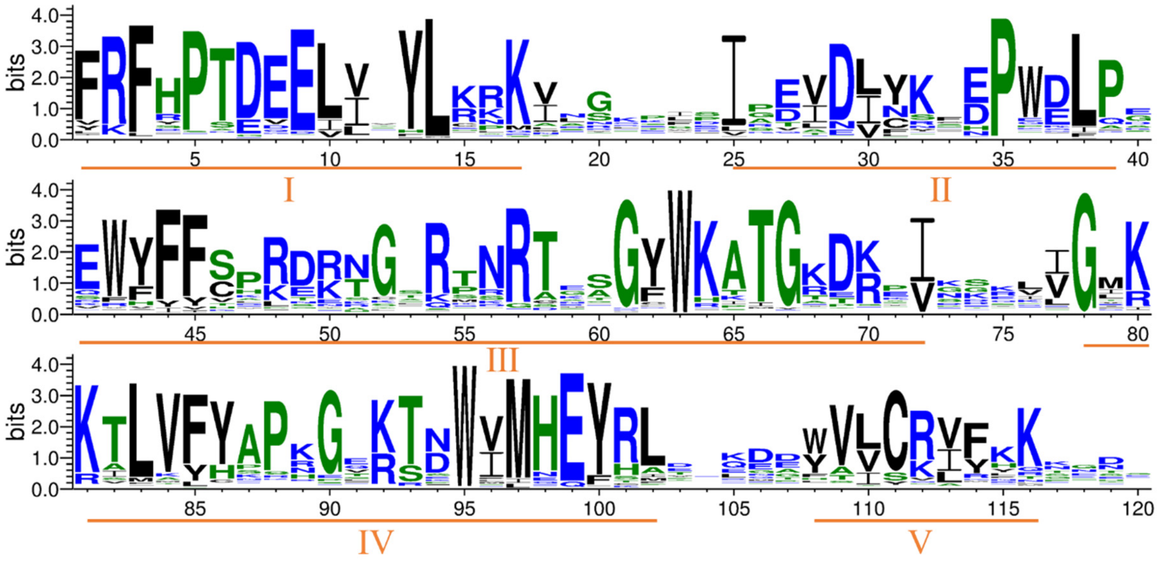

Figure. S3 Analysis for conserved motifs of NAC transcription factors.

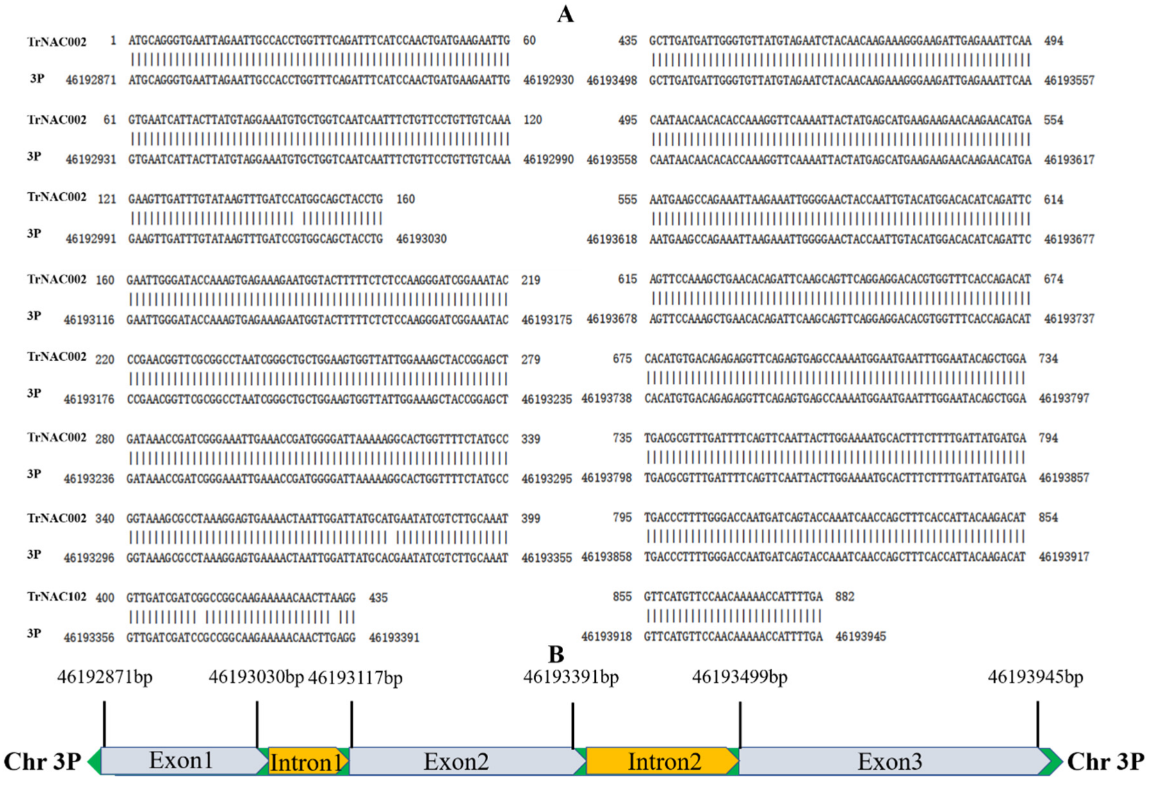

Figure. S4 Analysis for Chromosomal distribution, gene structure illustration of *TrNAC002*. A: aligning result of *TrNAC002* segments to chromosome 3P of white clover. B: illustration model of *TrNAC002* structure.

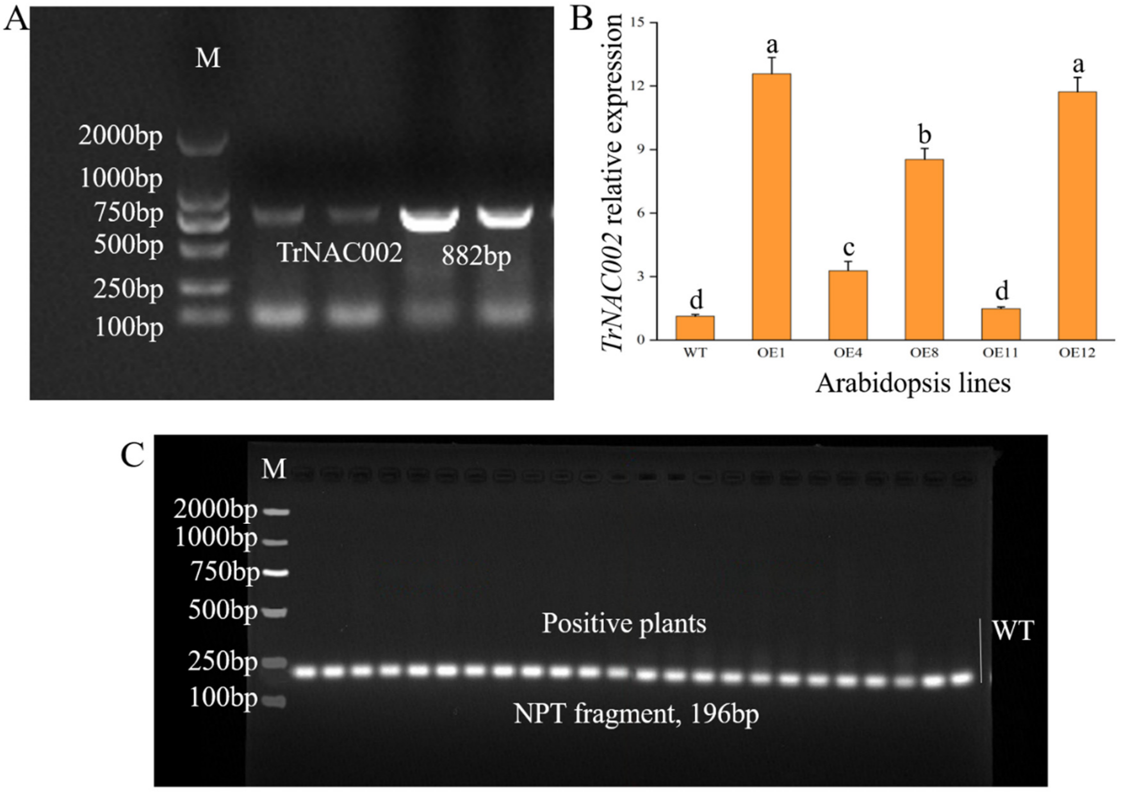

Figure. S5 Recombinant plasmid identification(A), relative expression of *TrNAC002* in *TrNAC002* overexpressing *Arabidopsis thaliana*(B), and PCR identification of positive plants

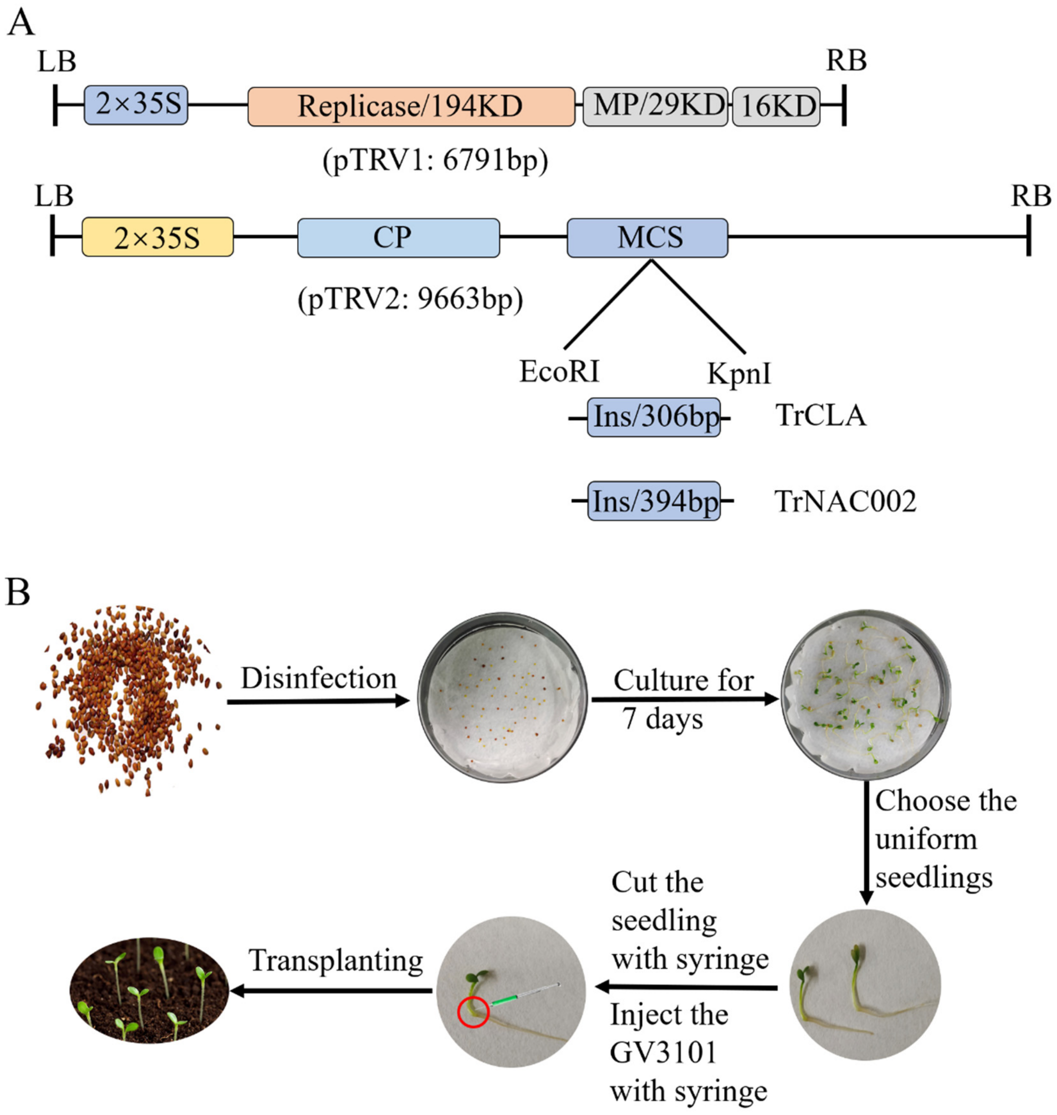

Figure. S6 Schematic representation of pTRV VIGS- system construction and TrCLA silencing in white clover. **A:** Genomic organization of the pTRV1 and pTRV2. Boxes indicate Open reading frames (ORF). The 306-bp fragment of TrCLA and the 394-bp fragment3 of *TrNAC002* are inserted in pTRV2. **B:** Illustration of infection experiment of white clover with mixture of pTRV1/GV3101 and pTRV2/GV3101.
